# PIPE-CLIP: a comprehensive online tool for CLIP-seq data analysis

**DOI:** 10.1186/gb-2014-15-1-r18

**Published:** 2014-01-22

**Authors:** Beibei Chen, Jonghyun Yun, Min Soo Kim, Joshua T Mendell, Yang Xie

**Affiliations:** 1 Quantitative Biomedical Research Center, University of Texas Southwestern Medical Center, Suite NC8.512 6000 Harry Hines Blvd, Dallas, TX 75390, USA; 2 Harold C. Simmons Comprehensive Cancer Center, University of Texas Southwestern Medical Center, Suite Nc8.512 6000 Harry Hines Blvd, Dallas, TX 75390, USA; 3 Department of Molecular Biology, University of Texas Southwestern Medical Center, 5323 Harry Hines Blvd, Dallas, TX 75390, USA

## Abstract

CLIP-seq is widely used to study genome-wide interactions between RNA-binding proteins and RNAs. However, there are few tools available to analyze CLIP-seq data, thus creating a bottleneck to the implementation of this methodology. Here, we present PIPE-CLIP, a Galaxy framework-based comprehensive online pipeline for reliable analysis of data generated by three types of CLIP-seq protocol: HITS-CLIP, PAR-CLIP and iCLIP. PIPE-CLIP provides both data processing and statistical analysis to determine candidate cross-linking regions, which are comparable to those regions identified from the original studies or using existing computational tools. PIPE-CLIP is available at http://pipeclip.qbrc.org/.

## Rationale

RNA’s diversity in sequence and structure endows it with crucial roles in cell biology [1]. Recent technological developments, especially the technique of crosslinking immunoprecipitation coupled with high-throughput sequencing (CLIP-seq), have provided powerful tools for studying the roles of RNA regulation in the control of gene expression and the generation of phenotypic complexity [1]. For example, high-throughput sequencing of RNA isolated by cross-linking immunoprecipitation (HITS-CLIP) was used to identify approximately 30 to 60 nucleotide regions around the peaks of CLIP read clusters that represent binding sites of RNA-binding proteins (RBPs) [2]. To increase detection sensitivity, photoactivatable-ribonucleoside-enhanced CLIP (PAR-CLIP) [1,3] was also developed. PAR-CLIP introduces photoactivatable ribonucleoside analogs, such as 4-thiouridine (4SU) and 6-thioguanosine (6SG), into the RNA of cultured cells to enhance cross-linking efficiency. This cross-linking process usually introduces mutations in sequence tags at RBP binding sites. For example, HITS-CLIP utilizes UV cross-linking of proteins with RNA, which introduces either insertions, deletions, or substitutions, depending on the RBPs [1,4]. PAR-CLIP introduces a distinct spectrum of substitutions (T-to-C for 4SU and G-to-A for 6SG). These cross-linking-induced mutations in HITS-CLIP and PAR-CLIP can be used as markers to identify the precise RBP binding sites. In addition, individual-nucleotide resolution CLIP (iCLIP) was developed to identify cross-linking sites independently of experimentally induced mutations. Instead, cDNA is circularized and then linearized at specific restriction sites, so that the truncation positions are used to locate candidate RBP binding positions [2,5].

Although several tools have been recently developed, there is still a lack of a comprehensive publicly available pipeline for analyzing CLIP-seq data. Piranha [6] is a tool mainly focusing on peak calling, without considering cross-linking-induced mutations. PARalyzer [7] and WavClusterR [8] are available as R packages for PAR-CLIP data analysis. PARalyzer estimates the likelihood of specific cross-linking-induced mutations, while wavClusterR uses wavelet transformation to distinguish between non-experimentally and experimentally induced transitions. Both tools, however, were developed only for PAR-CLIP data, and R packages may be inconvenient for experimentalists. A newly published tool, RIPseeker [9], is an R package based on a hidden Markov model for general RIP-seq experiment data analysis. It can process CLIP-seq data, but it does not utilize the specific characteristics of CLIP-seq data. Different from the tools mentioned above, CLIPZ [10] is an online web tool for analyzing CLIP-seq data with visualization functions. However, CLIPZ does not allow users to specify any analysis parameters. More importantly, it does not provide measurements of the statistical significance associated with specifically identified binding regions.

The aim of PIPE-CLIP is to provide a public web-based resource to process and analyze CLIP-seq data. It provides a unified pipeline for PAR-CLIP, HITS-CLIP and iCLIP, with the following features: (1) user-specified parameters for customized analysis; (2) statistical methods to reduce the number of false positive cross-linking sites; (3) statistical significance levels for each binding site to facilitate planning of future experimental follow-ups; and (4) a user-friendly interface and reproducibility features. PIPE-CLIP offers statistical methods that provide a significance level for each identified candidate binding site. Compared to the candidate cross-linking regions identified in the original studies for HITS-CLIP, PAR-CLIP and iCLIP, those identified by PIPE-CLIP are similar (using the cutoff based method) or slightly more reliable (using the statistics-based method). Furthermore, we demonstrate how different false discovery rate (FDR) cutoffs affect the number of identified candidate binding regions. Finally, we show that PIPE-CLIP has similar performance when identifying cross-linking regions from CLIP-seq data to other existing computational algorithms. This empirical study provides some guidance for users to select appropriate cutoff values for the analysis of novel datasets. In summary, PIPE-CLIP provides a user-friendly, web-based, ‘one-stop’ resource for the analysis of various types of CLIP-seq data.

## Materials and methods

### Pipeline overview

**Figure 1:**
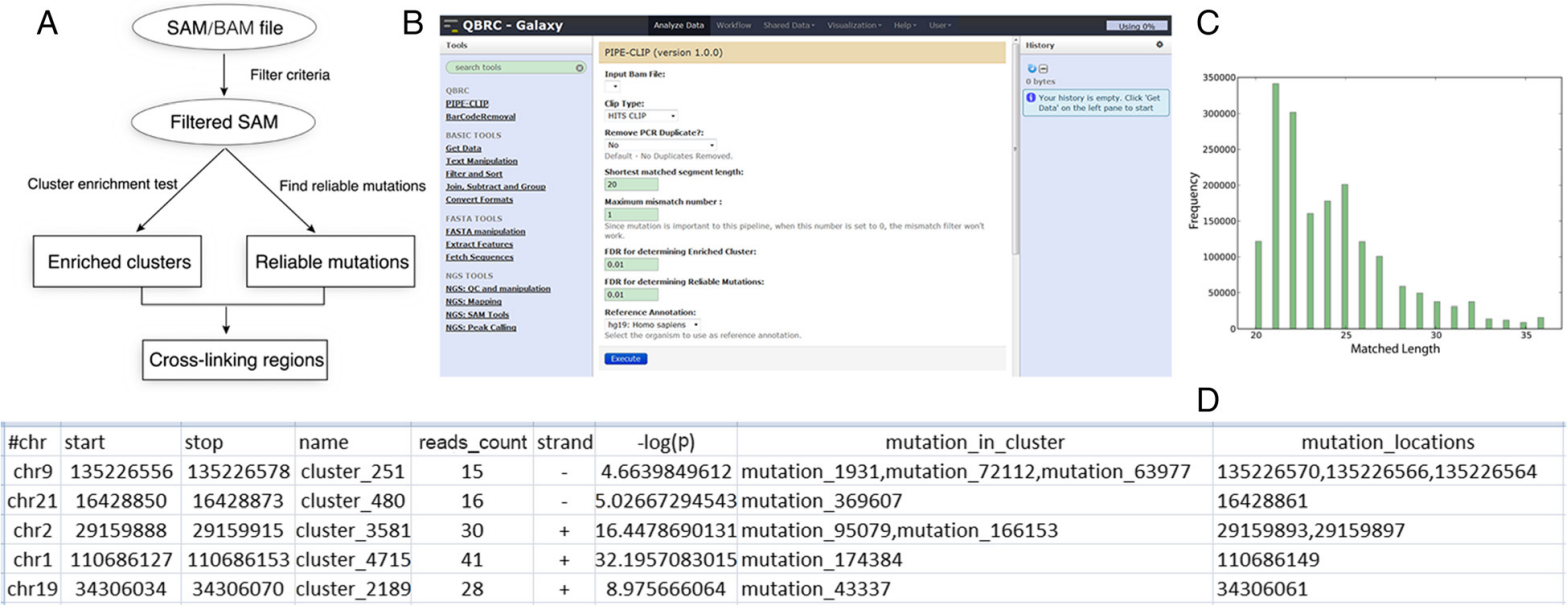
**PIPE-CLIP overview. (A)** Flowchart of PIPE-CLIP. Mapping results (in SAM/BAM format) are first filtered, and users then have an option to remove PCR duplicates. The filtered mapping files are then used to identify enriched clusters and reliable mutations. Each enriched cluster with at least one reliable mutation is then reported as a cross-linking region. **(B)** A screenshot of the PIPE-CLIP website. Users can upload SAM/BAM input files and perform customized data analysis by adjusting different parameters. Default parameters are provided based on our empirical experience. All of the parameters are automatically documented, so that the analysis procedure and results can be easily reproduced. A tool for removing PCR duplicates of iCLIP raw fastq data, according to specific barcodes, is also provided. **(C)** A sample output figure generated by running PIPE-CLIP reporting the length distribution of the mapped reads. **(D)** A demonstration of the output table for candidate cross-linking regions. The annotation of each column is detailed in the online user manual.

PIPE-CLIP identifies enriched clusters using sequence read counts, and pinpoints reliable binding sites using cross-linking-induced mutations (for PAR-CLIP and HITS-CLIP data) or cDNA truncation sites (for iCLIP data), and then combines both results to locate cross-linking regions (Figure 1). Procedures for data preprocessing and genomic annotation of the candidate regions are also included in the pipeline. Source code is available at [11].

### Data preprocessing

The PIPE-CLIP analysis pipeline accepts inputs in Sequence Alignment/Map (SAM) format or binary format (BAM) [12]. It preprocesses the data by filtering mapped reads and handling PCR duplicates. The main criteria for reads filtering are the minimum matched lengths and the maximum mismatch numbers for each read, and both parameters can be specified by users. Reads that meet both criteria are kept for further analysis. After the filtering step, users have different options to handle PCR duplicates. Based on the current literature for CLIP-Seq experiments [13-16], PCR duplicates are usually removed to avoid PCR artifacts, which in turn reduces the false positive rate in the identified cross-linking regions. However, removing duplicates may discard potentially good alignments and affect the results when the sequencing coverage is low [17]. Therefore, PIPE-CLIP allows users to decide whether to keep or remove PCR duplicates from the alignment file.

PIPE-CLIP users have an option to remove PCR duplicates using two different methods. The first method is based on the read start position and orientation, as described in Zhang *et al.*[4], while the second method takes sequence into account, along with mapping information. Specifically, the first method chooses a representative read from the cluster of reads that share the same starting genomic position, using the following sequential steps: (1) find the reads with the longest matched lengths; (2) find the reads with the fewest mismatch numbers; (3) find the reads with the highest quality scores; (4) choose one read randomly.

For the second approach, since the reads that map to the same position can still have different mutations, the reads are placed into groups by their sequences and steps 3 and 4 described above are executed, in order to find out the representative sequence for each group. For iCLIP data it is important to note that, since PCR duplicates are removed according to random bar codes before mapping, identical sequences in the SAM/BAM file represent real cDNA counts, and will not be removed in this step.

### Identifying enriched clusters

To identify enriched peaks, the adjacent mapped reads are clustered together if they overlap each other by at least one nucleotide, similar to ChIP-seq processing [18]. The clusters are used for further analysis. Let *r*_*i*_ denote the total number of reads within the *i*th cluster of length *s*_*i*_. Longer clusters tend to have greater read counts, so the variable *s*_*i*_ needs to be used to adjust the length effect on modeling *r*_*i*_. Given that all clusters receive at least one read, we propose a model equipped with the zero-truncated negative binomial (ZTNB) likelihoods.

We assume the ZTNB regression of *r* on *s* with the mean *μ*_*s*_ and the dispersion *θ*_*s*_^−1^. The ZTNB regression assumption yields the conditional density of *r* given *s* as:

(1) $$p\left(r|s,\mu_{s},\theta_{s}\right)=\frac{1}{1-p_{0}}\frac{\Gamma \left(r+\theta_{s}\right)}{\Gamma \left(\theta_{s}\right)\Gamma \left(r+1\right)}{\left(\frac{1}{1+\mu_{s}\theta_{s}^{-1}}\right)}^{\theta_{s}}{\left(\frac{\mu_{s}}{\theta_{s}+\mu_{s}}\right)}^{r},r>0,$$

where $p_{0}={\left(1+\mu_{s}\theta_{s}^{-1}\right)}^{-\theta_{s}}$ and *Γ*(⋅) is the gamma function. The length effect is incorporated into the model by link functions for *μ*_*s*_ and *θ*_*s*_ as follows:

$$\log\;\mu_{s}=\alpha +\log\;f\left(s\right)\mathrm{andlog}\;\theta_{s}=\beta +\log\;f\left(s\right),$$

where *f*(*s*) is used as an explanatory variable that represents the functional dependence of the read count on the cluster length. The link functions are slightly different from what has been typically used for the ZTNB regression model. In our model, we use *f*(*s*) instead of *s* as a predictor, so that the model is more general in the sense that the mean and variance function for *r* is allowed to be non-linear with respect to *s*. This model allows us to test whether a cluster is significantly enriched by reads, while adjusting the span of the cluster. For clusters of length *s*_*i*_ and read count *r*_*i*_, the *P*-value is defined as the probability of observing read counts ≥ *r*_*i*_. That is, the *P*-value = P(*r* ≥ *r*_*i*_|*s* = *s*_*i*_), where the probability law is derived from Equation 1.

For the model inference, first we estimate *f*(*s*) using the local liner regression [19] of *r* on *s*. Then, the estimate $\hat{f}\left(s\right)$ is plugged into the ZTNB regression as a predictor. To obtain maximum likelihood estimates (MLEs) of *α* and *β*, the conditional maximization method is implemented along with the Fisher’s scoring method [20] for *α* and the Newton-Raphson method for *β*. For more details about the model inference, please check the source code [21]. FDRs are calculated using the Benjamin-Hochberg procedure [22]. PIPE-CLIP reports the enriched clusters based on a user-specified FDR cutoff (the default is 0.01).

### Selecting reliable mutation/truncation sites

The identified cross-linking-induced mutations (for PAR-CLIP and HITS-CLIP) or cDNA truncations (for iCLIP) are clustered at each genomic location. For PAR-CLIP, only the characteristic mutations specified by users are included in the analysis. For HITS-CLIP, since cross-linking-induced mutations depend on the protein of interest, PIPE-CLIP processes substitutions, deletions and insertions separately, to allow the users to choose the type of cross-linking-induced mutation. For iCLIP, all of the cDNA truncations are included. Each location (one nucleotide) is characterized by two parameters (k_i_, m_i_), where k_i_ is the total number of mapped reads covering that location, and m_i_ is the number of specific mutations/truncations at location *i*. At each genomic location, m_i_ is modeled by a binomial distribution with size k_i_ and a success rate (that is, the reads coverage calculated using the sum of matched lengths of all reads that passed the filtering criteria in the data preprocessing step, divided by the genome size), and a *P*-value is calculated to assess the statistical significance of the mutation rate. Finally, FDRs are calculated from the *P*-values using the Benjamin-Hochberg method [22], and the locations with FDRs less than a user-specified cutoff are reported as reliable mutation/truncation sites.

### Identifying candidate cross-linking regions

Next, the identified reliable mutation/truncation sites are mapped to the enriched clusters. The enriched clusters (which passed the cluster FDR threshold) that contain reliable mutation/truncation sites (which passed the mutation/truncation FDR threshold) are reported as candidate cross-linking regions. We prioritize candidate cross-linking regions by combining the *P*-values using Fisher’s method [23]. Specifically, let *e*_*j*_ and *m*_*j*_ be the enriched cluster *P*-value and the smallest reliable mutation *P*-value of the *j*th candidate region, respectively. We define the *P*-value of the *j*th candidate region as:

$$c_{j}=P\left[\chi_{4}^{2}\geq -2\left(\log e_{j}+\log m_{j}\right)\right],$$

where *χ*_4_^2^ is a chi-square random variable with four degrees of freedom.

PIPE-CLIP generates one BED file, containing the candidate cross-linking regions for the characteristic mutations/truncation sites for PAR-CLIP and iCLIP data, while it also generates a BED file for each mutation type (substitution, deletion or insertion) separately for HITS-CLIP data.

### Annotating candidate cross-linking regions

Finally, the candidate cross-linking regions are annotated using the annotation package HOMER [24], which is a suite of tools for motif discovery and next-generation sequencing analysis, for the human (hg19/GRCh37.67) and mouse (mm10/GRCm38.69) genomes, providing information about the specific transcripts that are bound by the RBP of interest.

## Results and discussion

### PIPE-CLIP’s performance on PAR-CLIP data

PAR-CLIP sequencing data of three FET family proteins [17] was downloaded from the DNA Data Bank of Japan [DDBJ: SRA025082]. We mapped reads to the human genome (hg19) using Novoalign [25], and kept the uniquely mapped reads. To evaluate the performance of the PIPE-CLIP analysis, we compared the results from the PIPE-CLIP analysis with the original publication [17] and also checked whether the results were consistent with the biological expectation.

**Figure 2:**
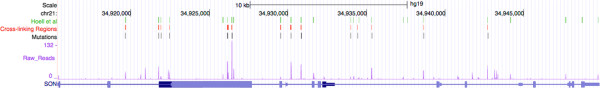
**FUS cross-linking regions within the gene *SON*.** The cross-linking sites found by Hoell *et al.*[17] and PIPE-CLIP; reliable mutations reported by PIPE-CLIP and read distributions within the *SON* gene body. The height of mutation bars represents the number of T-to-C mutations at specific locations (m value). The PIPE-CLIP analysis pipeline found 14 out of the 24 clusters that were identified by Hoell *et al.*[17]. Among the 10 clusters that were not identified by PIPE-CLIP, they did not have sufficient read coverage (10 reads) or did not contain any reliable mutation sites.

To compare the PIPE-CLIP analysis results with the original study [17], we first applied a cutoff-based approach using the same criteria: only clusters with ≥10 reads were considered, and at least 25% of the reads in an enriched cluster had to contain a T-to-C mutation to be considered a cross-linking region. A total of 41,468, 20,612 and 8,123 cross-linking regions for the FETS family proteins FUS, EWSR1 and TAF15, respectively, were found using the cutoff-based approach. This represents more cross-linking regions of FUS and EWSR1 and a similar count of TAF15 cross-linking regions compared to the results originally reported by Hoell *et al.*[17]. Next, we identified enriched clusters (based on the zero-truncated negative binomial model) and reliable mutations by applying different FDR thresholds implemented in PIPE-CLIP (Table 1). When using 0.01 as the FDR cutoff for both enriched clusters and reliable mutations, the numbers of identified cross-linking regions were 45,277, 16,470, and 7,038 for FUS, EWSR1 and TAF15, respectively. To compare results obtained using PIPE-CLIP with the findings of Hoell *et al.*, we examined specific genes with FET protein-binding sites identified in both analyses. For example, 24 PAR-CLIP clusters were previously identified within gene *SON* (chr21:34915350-34949812) [17]. The PIPE-CLIP analysis pipeline found 14 out of the 24 clusters using the statistical approach (Figure 2). Among 10 clusters that were not identified by PIPE-CLIP, eight did not have sufficient read coverage (<10 reads), and the remaining two clusters did not contain any reliable mutation sites (Figure 2). Therefore, we believe that the cross-linking regions identified by PIPE-CLIP are at least as reliable as the original study.

**Table 1:** Cross-linking regions identified by PIPE-CLIP for the FET family proteins data

**Number of cross-linking regions**	**FDR <0.1**	**FDR <0.05**	**FDR <0.01**	**FDR <0.001**	**FDR <0.0001**
EWSR1	43,311	31,601	16,470	12,154	11,205
FUS	59,880	53,847	45,277	37,322	34,576
TAF15	23,049	16,410	7,038	4,559	3,322

**Figure 3:**
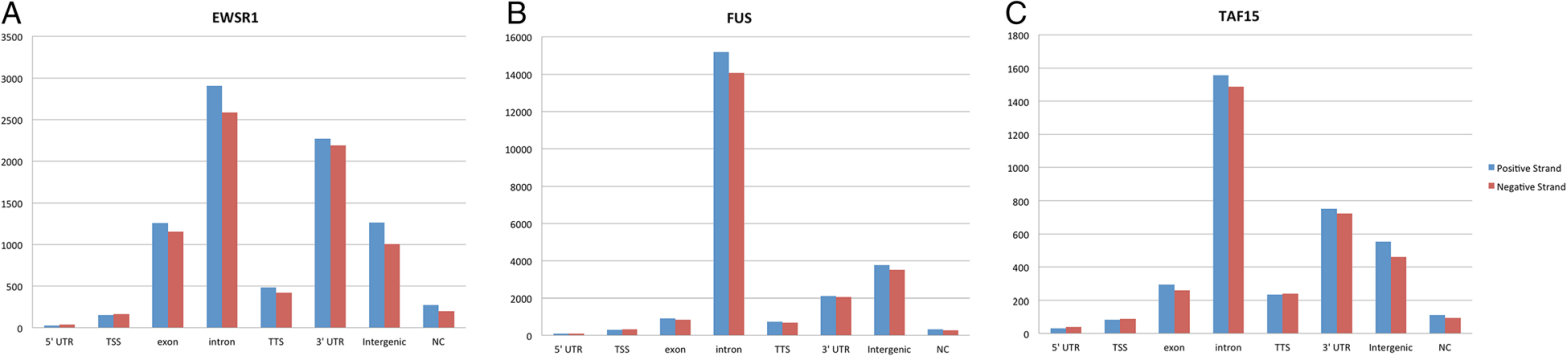
**Genomic annotation for cross-linking regions.** All of the candidate cross-linking regions of **(A)** EWSR, **(B)** FUS and **(C)** TAF15 identified by analyzing PAR-CLIP data using the negative binomial distribution analysis in PIPE-CLIP are annotated by HOMER (default parameters) [24]. The candidate cross-linking regions have similar genomic annotation distributions as reported by Hoell *et al.*[17] and the cross-linking regions are enriched in introns and 3’ UTRs. NC non-coding; TSSstands for transcription start site and TTS stands for transcription termination site.

**Figure 4:**
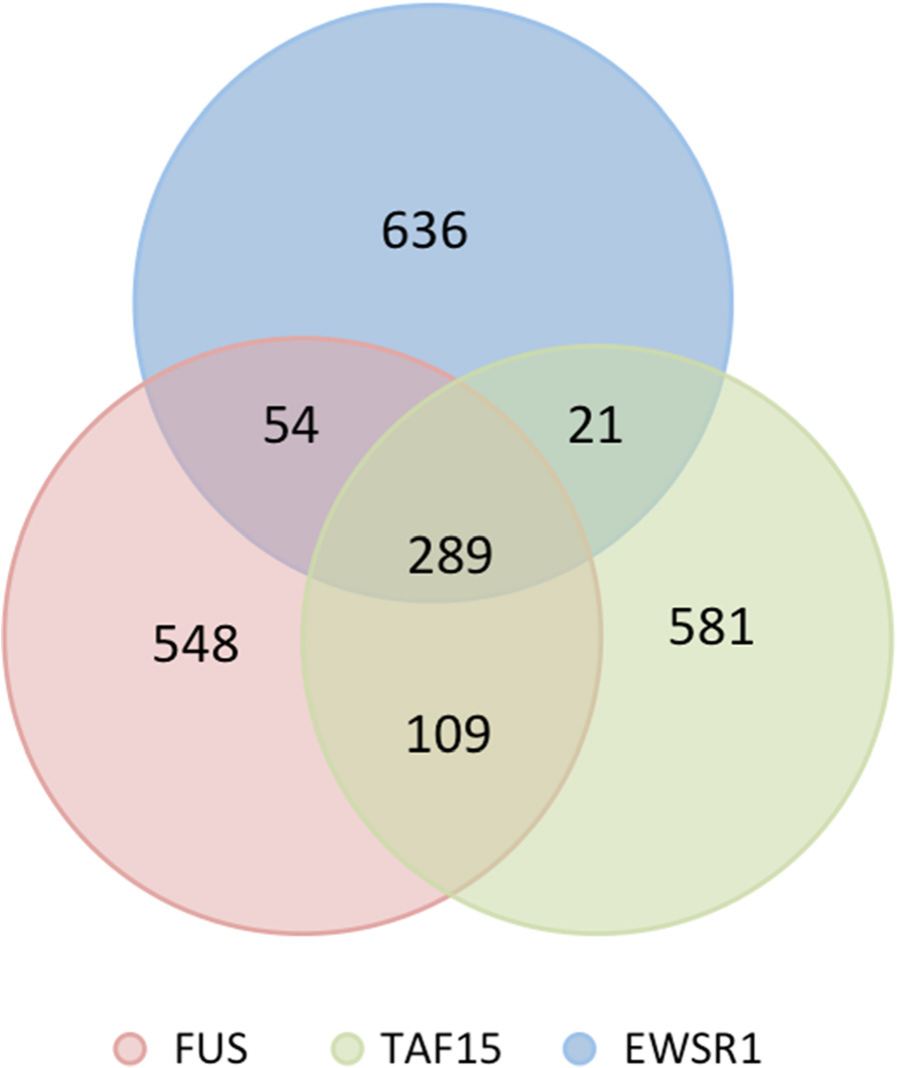
**Cross-linking region overlap among three FET family proteins.** Specific cross-linking regions of three FET family proteins (red, FUS; green, TAF15; blue, EWSR1) were ranked by their number of T-to-C mutations, and the top 1,000 regions for each protein were used for comparison. Two regions were considered overlapping when at least half of one region overlapped with another region.

To further evaluate whether the candidate cross-linking regions identified by the PIPE-CLIP approach were consistent with biological expectations, we checked the genomic annotations of the candidate regions (Figure 3) and the overlapping rates of the binding targets of the same three FET family proteins (Figure 4). Figure 3 shows that most of the cross-linking regions were within introns and 3’ UTRs, which is consistent with the biological expectation for this protein family [17]. Since EWSR1, FUS and TAF15 proteins are from the same protein family, considerable overlap among their binding sites is expected. To determine whether this is the case, the top 1,000 binding regions (identified by the zero-truncated negative binomial model and sorted by the number of reads in the regions) of the three proteins were compared (Figure 4). The results revealed significant overlap of binding regions among the FET proteins (hypergeometric test, *P*-value <1.5e-6), and the overlap frequencies were significantly higher than those reported in the original paper [17] (Fisher’s exact test; Table 2). Therefore, the analysis results from PIPE-CLIP are quite consistent with biological expectations.

**Table 2:** Comparison of the overlapping frequency of the 1,000 top enriched cross-linking regions of FET proteins identified in the original study versus by PIPE-CLIP software

	**Number of genes (Hoell *et al.*)**	**Number of genes (PIPE-CLIP)**	***P*-value (Fisher’s exact test)**
FUS overlap TAF15	332	398	0.003
FUS overlap EWSR1	239	343	1.885e-07
EWSR1 overlap TAF15	215	310	2.743e-06

### PIPE-CLIP’s performance on HITS-CLIP data

For HITS-CLIP analysis, Ago HITS-CLIP data for mouse brain was obtained from GSE16338 [26]. All the replicates were merged together and mapped to the mouse genome (mm10) using Novoalign [25], and only uniquely mapped reads were kept after removing duplicates. Basic parameters were the same as those described in Chi *et al.*[26]: a maximum of two-nucleotide mismatches were allowed, and a minimum match length of 25 nucleotides was required. We applied the different FDR cutoffs to the PIPE-CLIP algorithm, and the numbers of identified cross-linking regions as well as reliable deletions are shown in Table 3. Recently, Zhang and Darnell [4] proposed a computational approach, CIMS (crosslinking-induced mutation sites) analysis, to analyze HITS-CLIP data, which utilizes significant deletion sites to define cross-linking sites. PIPE-CLIP successfully identified 1,232 cross-linking regions when constrained to an FDR of 0.01 for both enriched clusters and mutations. Moreover, 398 of 886 CIMS mutations were covered by PIPE-CLIP cross-linking regions, while 834 cross-linking regions with significant deletions were identified by PIPE-CLIP, but not the CIMS algorithm.

**Table 3:** Cross-linking regions identified by PIPE-CLIP for the Ago HITS-CLIP data

	**FDR <0.1**	**FDR <0.05**	**FDR <0.01**	**FDR <0.001**	**FDR <0.0001**
Enriched clusters	58,614	41,390	20,781	8,744	6,288
Reliable mutations	14,957	14,271	5,872	5,546	5,044
Cross-linking regions	3,778	2,833	1,232	534	328

The total numbers of cross-linking regions identified by PIPE-CLIP using different FDR thresholds are shown. The FDR thresholds are the same for both the procedure of looking for enriched clusters and reliable mutations.

**Figure 5:**
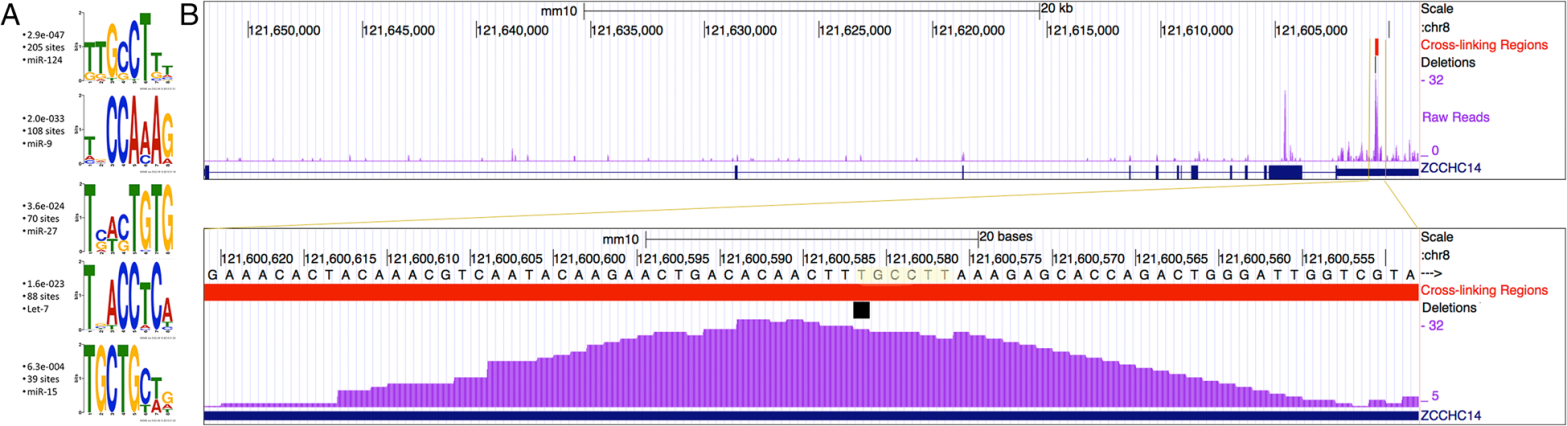
**Motif analysis and genomic location of Ago HITS-CLIP cross-linking regions. (A)** Motif analysis of identified candidate cross-linking regions. Flanking regions (−10 to +10 nucleotides) around identified reliable mutation sites within the cross-linking regions were used as input for the motif search tool MEME. All five motifs are complementary to microRNA seed regions. **(B)** Example of an miR-124 binding site within the transcript of *Zcchc14*. Top panel: reads enriched in the 3’ UTR region of *Zcchc14*. Bottom panel: a zoomed-in view of the candidate cross-linking region. The highlighted nucleotides correspond to the seed-binding region of miR-124.

To further evaluate the performance of PIPE-CLIP in identifying binding sites, the flanking regions (-10 nucleotides, +10 nucleotides) of all deletion sites within candidate cross-linking regions (FDR <0.01) were used to search for significant motifs (using the motif-searching tool MEME). All of the significant motifs (e < 1), except the polyA motif (AAUAAA), were associated with specific microRNAs (Figure 5A). Among these five motifs, four (the seed-binding motifs of miR-124, miR-9, miR-27 and let-7) were also reported as the significant microRNA seeds by the CIMS analysis [4], while the seed-binding motif of miR-15, which was reported to be associated with Argonaute (Ago) in mouse brain [27], was identified only by PIPE-CLIP. Figure 5B shows an example of a miR-124 binding site within *Zcchc14* (chr8:121598703-121651933). These results indicate that the cross-linking regions identified by PIPE-CLIP are highly reliable in predicting microRNA-binding motifs.

### PIPE-CLIP’s performance on iCLIP data

iCLIP sequencing data for the RBP Nova was downloaded from ArrayExpress [ArrayExpress:E-MTAB-1008]; PCR replicates were removed according to their barcodes. Next, the barcodes were removed, and the reads were mapped to the mouse genome (mm10), using the same parameters as described above. For iCLIP experiments, truncation sites can represent the majority of the cross-linking sites, and have been used in the analysis [28]. Table 4 summarizes the number of enriched clusters and truncation sites when using different FDR thresholds in PIPE-CLIP. Since the specific number of Nova iCLIP truncation sites was not mentioned in the original paper, we did not compare our list with theirs.

**Table 4:** PIPE-CLIP results summary for the Nova iCLIP data

**FDR**	**<0.1**	**<0.05**	**<0.01**	**<0.001**	**<0.0001**
Number of enriched clusters	7,837	4,283	1,956	1,059	775
Number of reliable truncations	4,724	4,584	3,974	953	851
Number of cross-linking regions	3,861	2,153	848	376	288

The total numbers of enriched clusters, reliable truncations and cross-linking regions identified by PIPE-CLIP using different FDR thresholds are shown. The FDR threshold is the same for both the procedure of looking for enriched clusters and reliable mutations.

**Figure 6:**
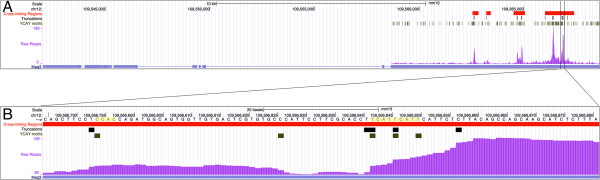
**Nova binds to *Meg3*. (A)** Overview of cross-linking regions (red track) of Nova protein found by PIPE-CLIP within the *Meg3* gene (chr12:109542023-109568594, mm10). Most of the reads localized to the 3’ terminal exon. In total, there were four enriched clusters within this region, and most of the identified reliable truncations (black track) were located close to the enriched regions’ summits. **(B)** A zoomed-in view of the candidate cross-linking region. The YCAY motifs and truncation sites are highlighted.

It is well known that Nova-binding regions are enriched for YCAY motifs [29-34]. In order to check whether the Nova binding regions found by PIPE-CLIP also contained this motif, all of the reliable truncation positions within cross-linking regions (FDR <0.01 for both enriched clusters and reliable truncations) were extended 10 nucleotides at both the 5’ and 3’ ends. Out of 1,017 truncation regions, 370 contain YCAY motifs. We also checked the *MEG3* gene (chr12:109542023-109568594), which is a maternally expressed non-coding RNA and a primary target of Nova binding [28], for the YCAY motif. As shown in Figure 6, PIPE-CLIP successfully identified cross-linking regions in the 3’ terminus of MEG3 (top panel), with most truncation sites having an YCAY motif right to them (highlighted in the bottom panel). These results are similar to the original publications and are consistent with the biological expectations.

### Comparing PIPE-CLIP’s performance with other computational tools

Recently, several computational tools were developed for analyzing PAR-CLIP data. Using the FET family protein data described above, we compared PIPE-CLIP’s performance with published computational tools, including Piranha [6], PARalyzer [7] and MACS2 [35]. Piranha is a universally peak caller for CLIP-seq and RIP-seq data that bins all the mapped reads according to their starting point on the genome. The total reads counted in the bin, together with some other covariates such as mappability, are used to fit a certain (user defined) distribution model to determine whether a specific bin is enriched or not. For this analysis, a negative binomial distribution was selected since it generally has good performance and is matched with the distribution used in PIPE-CLIP. MACS2 is a popular peak caller for ChIP-seq data, but it is also used in various other high-throughput sequencing data for peak calling purposes. The MACS2 models peaks on positive strands and negative strands based on a Poisson distribution [35]. After that, peaks from positive and negative strands are paired and moved in the 3’ direction until their middle points are at the same position, and that position is then reported as a peak summit. The default parameters of MACS2 were used to generate results. PARalyzer is a computational algorithm designed for PAR-CLIP data. It groups adjacent mapped reads and generates two smoothened kernel density estimates within each read group, one for T-to-C transitions and one for non-transition events. Nucleotides within the read groups that maintain a minimum read depth, and where the likelihood of T-to-C conversion is higher than non-conversion, are considered interaction sites. Again, we implemented the default parameters in the PARalyzer package to identify cross-linking regions for the three FET family proteins.

**Figure 7:**
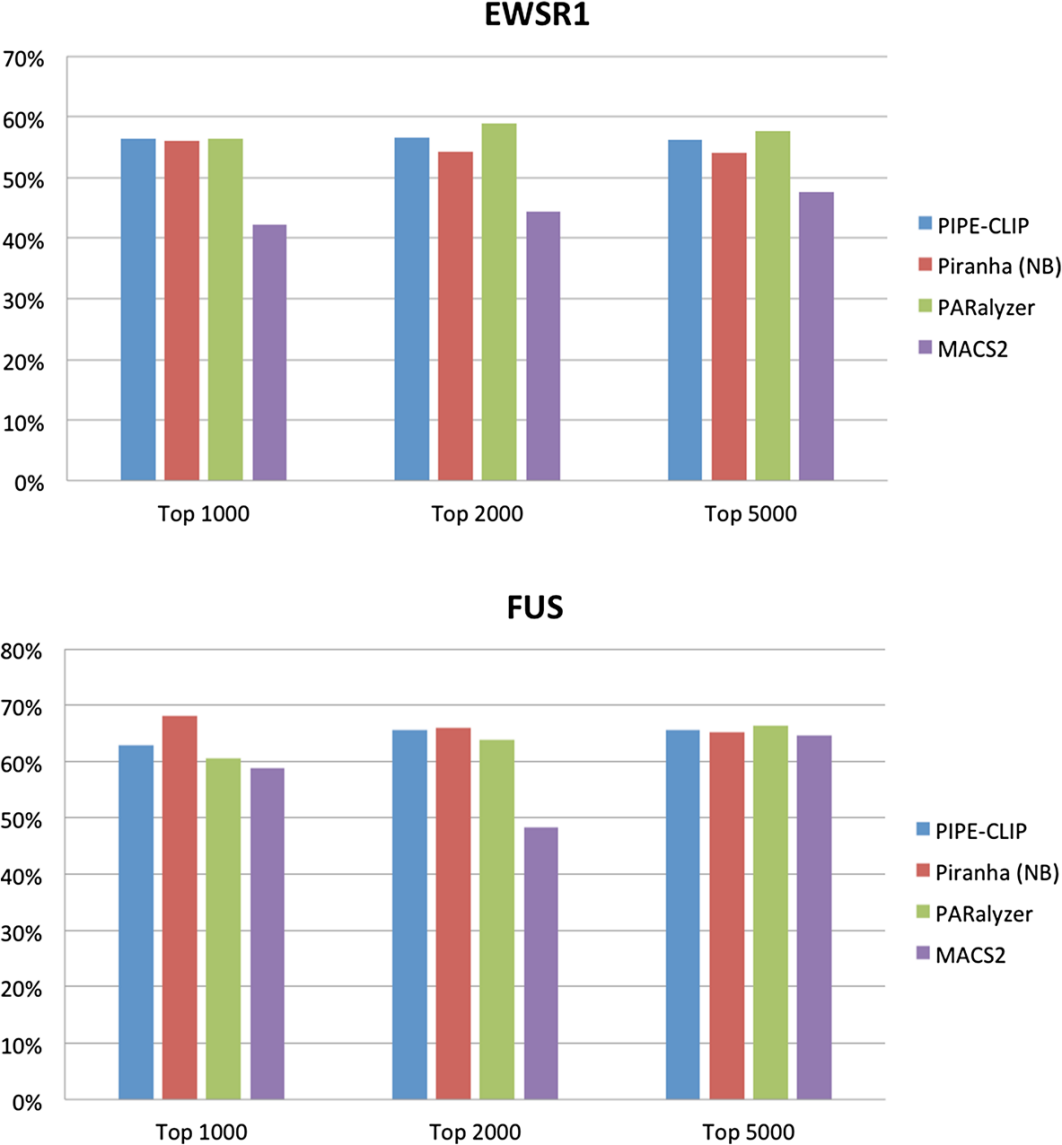
**Comparison with other computational tools for analyzing PAR-CLIP data.** The reliable target gene lists for EWSR1 and FUS proteins were obtained from an independent study [36] and used as a standard for comparison of different computational tools. Cross-linking regions are sorted according to the read counts in that region, and the top 1,000, 2,000, and 5,000 regions were selected as the candidate binding regions from each computational method. The percentage of coverage (y-axis in the figure) was defined as the ratio of the number of selected cross-linking regions that were covered in the reliable gene regions (5' UTR to 3' UTR of a gene in the reliable target list) compared to the number of selected cross-linking regions that were covered in the 5' UTR to 3' UTR of any genes in the genome. We calculated the percentages of coverage for PIPE-CLIP, Piranha, PARalyzer and MACS2 for both EWSR1 and FUS proteins.

To evaluate the performance of these four different computational tools, we obtained the lists of target genes of FUS and EWSR1 proteins from an independent study published by Han *et al*. [36]. In that study, biotinylated isoxazole (b-isox) was used to form RNA granule-like aggregates in cell lysates to co-immunoprecipitate proteins and their bound RNAs. The relative abundances of these RNAs in the control and the knockdown conditions were used to determine the binding strength of the RBP to its gene targets [36]. We obtained lists of genes that contained reliable FUS and EWSR1 binding sites (score <0.95) from that particular study [36]. All the cross-linking regions were ranked by the read numbers in each region and the top 1,000, 2,000 and 5,000 regions selected by PIPE-CLIP, Piranha, PARalyzer and MACS2 were selected and compared to the target gene lists to see how many of them comprised the gene region. Figure 7 shows that PIPE-CLIP, Piranha, and PARalyzer outperformed MACS2, which was not designed for CLIP-seq or RIP-seq data, and PIPE-CLIP, Piranha and PARalyzer all exhibited similar performance. Therefore, we conclude that PIPE-CLIP has comparable performance in identifying binding targets for PAR-CLIP data to the other three computational tools.

Currently, there exist few computational tools to analyze HITS-CLIP or iCLIP data. PARalyzer was designed for PAR-CLIP data analysis, and MACS2, designed for ChIP-seq data, does not consider mutation or truncation information. We thus implemented the Piranha algorithm for Ago HITS-CLIP data and Nova iCLIP data, but it could not identify any binding targets using a FDR cutoff of 5%. As shown in the previous results, PIPE-CLIP identified reasonable cross-linking regions using the same FDR cutoff. In addition, we also performed simulation studies and showed that PIPE-CLIP performed better than CIMS in the simulation studies (Additional file 1).

## Conclusions

PIPE-CLIP is a web-based resource designed for detecting cross-linking regions in HITS-CLIP, PAR-CLIP and iCLIP data. It is based on a Galaxy open-source framework, and accepts SAM/BAM format as input. It reports cross-linking regions with high reliability. Comparative analysis with several publicly available data sets and several existing computational tools showed that PIPE-CLIP has a performance comparable with other methods for identifying cross-linking sites from CLIP-seq experiments. Users can easily tailor different parameters for processing steps and choose statistical thresholds for identifying candidate binding sites, and compare all the results. All such user-specified parameters are well documented, and the intermediate outputs provided, in order to make it convenient for users to trace back the analysis steps. Details of usage are available online. A script (barcodeRemover) to remove barcode and PCR duplicates for iCLIP is also provided at the same website [37]. In conclusion, PIPE-CLIP provides a comprehensive, user-friendly and reproducible analytical resource for various types of CLIP-seq data.

## Abbreviations

4SU: 4-thiouridine; 6SG: 6-thioguanosine; CIMS: crosslinking-induced mutation sites; CLIP: cross-linking immunoprecipitation; CLIP-seq: cross-linking immunoprecipitation coupled with high-throughput sequencing; FDR: false discovery rate; HITS-CLIP: high-throughput sequencing of RNA isolated by cross-linking immunoprecipitation; iCLIP: individual-nucleotide resolution CLIP; PAR-CLIP: photoactivatable-ribonucleoside-enhanced CLIP; PCR: polymerase chain reaction; RBP: RNA-binding protein; UTR: untranslated region; ZTNB: zero-truncated negative binomial.

## Competing interests

The authors declare that they have no competing interests.

## Authors’ contributions

BC, JY and YX designed the project and developed the underlying algorithms. BC and JY wrote pipeline code, performed the testing and analysis and wrote the online user guide. MK set up the Galaxy interface. All authors together wrote the manuscript. All authors read and approved the final manuscript.

## Supplementary Material

Additional file 1 Supplement to ‘PIPE-CLIP: a comprehensive online tool for CLIP-seq data analysis’. Click here for file
