# Supplementary material for: PIPE-CLIP: a comprehensive online tool for CLIP-seq data analysis
Source: Genome Biol. 2014 Jan 22;15(1):R18. doi: 10.1186/gb-2014-15-1-r18 (PMC4054095; doi:10.1186/gb-2014-15-1-r18)
Supplement: Additional file 1 — Supplement to ‘PIPE-CLIP: a comprehensive online tool for CLIP-seq data analysis’. [file gb-2014-15-1-r18-S1.pdf]

Supplement to “PIPE-CLIP: a comprehensive online tool for CLIP-seq data analysis”

Beibei Chen, Jonghyun Yun, Min Soo Kim, Joshua T. Mendell and Yang Xie

Simulation Studies

We compare performance of our method with the CIMS analysis (Zhang and Darnell, 2011). In the CIMS, a permutation method is adopted to identify crosslinking mutation sites. In the permutation procedure, each mutation is planted into randomly selected reads while keeping the offset from the 5’ end of original reads. Then, cumulative numbers of sites with mutations $\leq x$ at read count r are used to estimate the false discovery rate (FDR). Our method and the CIMS with 5 repeated permutations are implemented in the artificial data. Read sequences are generated using mixture models, and mutations are simulated to accommodate the CIMS that utilizes more features in mutations.

We generate 50,000 regions for each independent experiment. In each region, 18 consecutive genomic locations are bases to generate reads. These 18 locations are divided into 3 disjoint blocks of 6 consecutive sites. Then, 5% of blocks are randomly chosen as enriched peaks. Let $\nu_i^{[t]}$ denote the number of reads generated at the t-th location of cluster i. We draw $\nu_i^{[t]}$ from the negative binomial with a size 50 and a mean $\frac{\varsigma_i^{[t]}}{1-\varsigma_i^{[t]}}$ where $\operatorname{logit}\varsigma_i^{[t]}$ follows the mixture normal distribution as

$$\operatorname{logit}\varsigma_i^{[t]} \sim \begin{cases} \mathrm{N}(1, 0.5^2) & \text{if the site is in enriched peaks;} \\ \mathrm{N}(6.75, 0.5^2) & \text{o.w.,} \end{cases}$$

where $\mathrm{N}(\mu, \sigma^2)$ denotes the normal distribution with the mean μ and the variance σ^2. Then, distances to start and end of reads from each base are chosen from the uniform distribution on $\{8, 9, \ldots, 22\}$, and each cluster is extended according to read-sequences generated.

True and error mutations are planted in read sequences, while first and last five positions in read sequences and all positions in reads whose lengths are less than 26 are set to be in the zero state that receives no mutations. A half of enriched peaks are randomly chosen as crosslinking regions. At each of the chosen enriched peaks, one site out of 6 sites is randomly selected as the crosslinking mutation site. For these crosslinking mutation sites, mutation probabilities $\rho_i^{[t]}$'s are drawn from $\mathrm{Ba}(a, b)$ which denotes the beta distribution with a mean $a(a+b)^{-1}$ and a variance $ab(a+b)^{-2}(a+b+1)^{-1}$. Then, mutations are planted with probability $\rho_i^{[t]}$ on the collection of non-zero state positions that are mapped into each crosslinking mutation site. Error mutations are planted on every g-th position in reads of length l with error mutation probabilities $\vartheta_{l,g}$'s which are increasing with respect to g. The sum of the error probabilities $\sum_{g=6}^{l-5} \vartheta_{l,g}$ is an increasing function of l, which is upper-bounded at 1.

Our method and the CIMS are implemented on the artificial data. The CIMS reports FDRs for each site, and minimum FDR values in each cluster are assigned as FDRs to corresponding clusters. In both methods, clusters with large reads and mutations are considered as crosslinking regions. The predictive power of each method to identify crosslinking regions is measured by the areas under the ROC curves (AUCs). The experiment is repeated 100 times for six pairs of (a, b). Means of beta distributions are 0.1 for $(a, b) \in \{(.5, 4.5), (1, 9), (2, 18)\}$ and 0.2 for $(a, b) \in \{(1, 4), (2, 8), (4, 16)\}$, respectively. In each mean level, pairs are arranged by variances in descending order. The average AUCs and the average differences of AUCs between the two methods are presented in Web Table **??**.

In the CIMS, the effectiveness of identification relies on clustered mutations on the crosslinking mutation site. If non-crosslinking and crosslinking mutation sites cannot be well separated by clustered mutations, the CIMS may lose some effectiveness. In the mean while, our method utilizes the distribution of cluster read counts along with clustered mutations, so that the loss of the identification efficiency due to the poor separability would be lessen in our method. Web Table **??** shows that our method outperforms the CIMS in all six settings. Means of AUC differences between the two methods are

Web Table 1: Average AUCs and their standard errors of the PIPE-CLIP (top), the CIMS (bottom) and differences of the two methods (middle) based on clusters. Six pairs of parameters are used for Beta distributions to draw probabilities to generate true mutations.

	Beta(.5,4.5)	Beta(1,9)	Beta(2,18)
PIPE-CLIP	.9556 (.0001)	.9642 (.0001)	.9768 (.0001)
Difference	.0237 (.0001)	.0217 (.0001)	.0185 (.0001)
CIMS	.9319 (.0001)	.9426 (.0001)	.9583 (.0001)
	Beta(1,4)	Beta(2,8)	Beta(4,16)
PIPE-CLIP	.9765 (.0001)	.9907 (.0001)	.9970 (.0001)
Difference	.0190 (.0001)	.0112 (.0001)	.0062 (.0001)
CIMS	.9575 (.0001)	.9795 (.0001)	.9908 (.0001)

greater for settings with smaller means and larger variances of mutation to read ratios. As the mean of mutation to read ratios increases, the CIMS works as effectively as our method (not presented here). Overall, our method performs better than the CIMS when crosslinking mutation sites have small mutation to read ratios. Under large variances of the ratios, the CIMS loses more predictive power than our method.
